# Genetic control of *Aedes aegypti*: data-driven modelling to assess the effect of releasing different life stages and the potential for long-term suppression

**DOI:** 10.1186/1756-3305-7-68

**Published:** 2014-02-13

**Authors:** Peter Winskill, Angela F Harris, Siân A Morgan, Jessica Stevenson, Norzahira Raduan, Luke Alphey, Andrew R McKemey, Christl A Donnelly

**Affiliations:** 1 Department of Infectious Disease Epidemiology, Medical Research Council Centre for Outbreak Analysis and Modelling, Faculty of Medicine, Imperial College London, St Mary’s Campus, London, UK; 2 Oxitec Limited, Oxford, UK; 3 Mosquito Research and Control Unit, George Town, Cayman Islands; 4 Vector Group, Liverpool School of Tropical Medicine, Liverpool, UK; 5 Institute for Medical Research, Jalan Pahang, 50588 Kuala Lumpur, Malaysia; 6 Department of Zoology, University of Oxford, Oxford, UK

**Keywords:** *Aedes aegypti*, Dengue, RIDL, Transgenic, Sterile insect technique, SIT, Pupae

## Abstract

**Background:**

Control of the world’s most important vector-borne viral disease, dengue, is a high priority. A lack of vaccines or effective vector control methods means that novel solutions to disease control are essential. The release of male insects carrying a dominant lethal (RIDL) is one such approach that could be employed to control *Aedes aegypti*. To maximise the potential of RIDL control, optimum release strategies for transgenic mosquitoes are needed. The use of field data to parameterise models allowing comparisons of the release of different life-stages is presented together with recommendations for effective long-term suppression of a wild *Ae. aegypti* population.

**Methods:**

A compartmental, deterministic model was designed and fitted to data from large-scale pupal mark release recapture (MRR) field experiments to determine the dynamics of a pupal release. Pulsed releases of adults, pupae or a combination of the two were simulated. The relative ability of different release methods to suppress a simulated wild population was examined and methods to maintain long-term suppression of a population explored.

**Results:**

The pupal model produced a good fit to field data from pupal MRR experiments. Simulations using this model indicated that adult-only releases outperform pupal-only or combined releases when releases are frequent. When releases were less frequent pupal-only or combined releases were a more effective method of distributing the insects. The rate at which pupae eclose and emerge from release devices had a large influence on the relative efficacy of pupal releases. The combined release approach allows long-term suppression to be maintained with smaller low-frequency releases than adult- or pupal-only release methods.

**Conclusions:**

Maximising the public health benefits of RIDL-based vector control will involve optimising all stages of the control programme. The release strategy can profoundly affect the outcome of a control effort. Adult-only, pupal-only and combined releases all have relative advantages in certain situations. This study successfully integrates field data with mathematical models to provide insight into which release strategies are best suited to different scenarios. Recommendations on effective approaches to achieve long-term suppression of a wild population using combined releases of adults and pupae are provided.

## Background

The mosquito-borne viral infection, dengue, has shown trends of increasing incidence [1] and geographical distribution [2,3] in recent years. It is now estimated that there are 390 million new infections every year with symptoms ranging from mild influenza-like illness to death [1,3]. Currently there is no vaccine or specific case treatment for dengue fever or the associated dengue haemorrhagic fever (DHF) and dengue shock syndrome (DSS). Control of *Aedes spp*, the mosquito vectors of the virus, remains the primary approach in the prevention of dengue. However, there is a lack of effective tools for controlling the vector, emphasising the importance of new methods of control.

The release of insects carrying a dominant lethal (RIDL) is one such approach. Engineering mosquitoes that are functionally genetically sterile, sex-sorting and releasing males to compete with wild males for a wild female mate is a modern and more tractable alternative to the traditional sterile insect technique (SIT) [4,5]. The technology has been used to successfully demonstrate control of a wild population of *Ae. aegypti* in the Cayman Islands [6].

Efficient implementation of the RIDL approach in the field relies on effective release methods. Previous trials have released male mosquitoes at both the pupal and adult life stage [6,7]. Adult-only releases are common in SIT control programmes but releases at earlier life stages have also been documented [8,9]. An alternative approach is a combined release in which both adults and pupae are released. To inform the design of control programmes there is a need for a better understanding of the comparative dynamics and performance of pupal-only and combined releases relative to adult-only releases.

Modelling studies may serve a critical role in the optimisation of control programmes, allowing many potential approaches to be investigated at the theoretical level. In order for such studies to be accurate and applicable, it is vital that they are well informed with appropriate field data. In this study, data from a large-scale field study provide the foundation for a model describing the dynamics of a pupal release. The work goes on to use the data-driven model to investigate the dynamics of adult-only, pupal-only and combined RIDL releases, with the specific aim of highlighting potential benefits of pupal-only or combined releases relative to adult-only releases. Different release methods and regimes are simulated and their ability to suppress a simulated wild population analysed. A successful vector control programme must be sustainable in the long-term. Furthermore, it must be able to withstand perturbations in wild population densities induced through immigration of wild individuals into the target population from neighbouring high-density or uncontrolled areas. Such immigration pressures have the potential to seriously hinder a sterile-insect approach [10] and are therefore an important aspect to consider. Potential approaches that take advantage of releasing different life stages to maintain long-term population suppression in the presence of mosquito immigration into the control area are shown.

## Methods

### Data

Pupal MRR data were collected during a field trial in Grand Cayman, a British Overseas Territory in the Caribbean, between September and October 2010 [6]. The study location was a peri-domestic area consisting mainly of mixed brick and wooden housing [7]. Pupae (strain: OX513A) were distributed in pupal release devices that were placed in shaded locations across the study site. The release device consisted of two stacked deli pots (base diameter = 9 cm, top diameter = 10 cm, base pot height = 7 cm, upper pot height = 3.5 cm). The lower pot housed the pupae in approximately 2 cm of water. The upper pot consisted of an open meshed base and lid and was filled with polystyrene beads coated with the fluorescent dust. The device was placed in a water ant-trap to minimise the risk of predation. Pupae eclose within the device where the resultant adult males may rest before being marked with fluorescent dust whilst exiting the device through the matrix of polystyrene balls [11]. During four independent MRR experiments, 19624, 38968, 16673 and 22702 pupae were released at the field site. Recaptures were made on subsequent days using 15 BG-Sentinel traps (Biogents) distributed across the study area. It is assumed, as observed in preliminary data, (not shown), that marked individuals do not transfer markings to unmarked individuals.

Pupal eclosion experiments were performed simultaneously to pupal MRR. Pupae were sampled from the release generation and placed in small cages (25 × 25 × 25 cm). Cages were stored overnight at 20°C and then transferred to an outdoor, semi-shaded location. The number of individuals that had eclosed was recorded at subsequent 24-hour intervals until eclosion ceased. Five replicates of the pupal eclosion experiment were conducted with 230, 252, 274, 292 and 267 individuals each.

### Release model

A continuous-time compartmental deterministic model was developed to fit to pupal MRR data. The rate of change in the number of male pupae (P) decays logistically with respect to time

(1) $$\frac{\mathrm{dP}}{\mathrm{dt}}=-\left(\frac{\mathrm{wP}\left(k-P\right)}{k}\right),$$

where *w* is the pupal eclosion rate and *k* a pupal coefficient producing a sigmoidal decay in pupae numbers over time. The model assumes that all pupae successfully eclose, all individuals are homozygous males and that males exit the release device immediately. Pupae can progress to being sexually immature newly eclosed adults (A). These individuals remain sexually immature for an average of σ^-1^ days. Sexually immature adults may die or be recaptured with rates δ and γ, respectively. The mortality term (δ) encapsulates both true mortality plus emigration from the study area. The size of the study area should minimise emigration effects. The rate of change in the number of sexually immature adults (A) with respect to time is given

(2) $$\frac{\mathrm{dA}}{\mathrm{dt}}=\left(\frac{\mathrm{wP}\left(k-P\right)}{k}\right)-\mathrm{\sigma A}-\mathrm{\gamma A}-\mathrm{\delta A}.$$

Those adults that have survived to sexual maturity are assumed to die or be recaptured with the same time-independent rates as sexually immature adults δ and γ, respectively. The rate of change in the number of sexually mature adults (M) with respect to time is

(3) $$\frac{\mathrm{dM}}{\mathrm{dt}}=\mathrm{\sigma A}-\mathrm{\gamma M}-\mathrm{\delta M}.$$

The rate of change in the number of recaptured (R) individuals is therefore dependent on the rate of recapture of both sexually immature and mature adults

(4) $$\frac{\mathrm{dR}}{\mathrm{dt}}=\mathrm{\gamma A}+\mathrm{\gamma M}.$$

A model with an additional compartment, representing eclosed adults that had not left the pupal release device, was also considered (Additional file 1: Appendix 1). The adult model used to simulate adult-only releases is a simplification of Equation 3, where *A* = 0. Adult males are released when sexually mature and are assumed to die or be recaptured with rates δ and γ, respectively, equal to those of adults in a pupal release. The rate of change in the total number of adult individuals with respect to time is

(5) $$\frac{\mathrm{dM}}{\mathrm{dt}}=-\mathrm{\gamma M}-\mathrm{\delta M}.$$

Pulsed releases can be simulated by summing multiple instances of single releases across release time points. The pupal model (Equations 1, 2 and 3), parameterised from field data, alongside a more simple adult-only release model (Equation 5) was used to simulate pulsed releases of RIDL insects. Releases could be adult-only, pupal-only or combined.

### Parameterisation of the model

Model fitting and parameter estimation were performed, using Berkeley Madonna [12], by minimising the sum of squared differences between model-predicted recapture estimates (Equation 4) and the observed recapture data from four pupal MRR experiments. Data describing pupal eclosion rates in the field allowed comparison of observed versus expected pupal eclosion rates from the best fit models as a means of validation. Poisson 95% confidence intervals were calculated based on recapture data. Confidence intervals surrounding eclosion data were calculated using the product of variance from both eclosion and release data.

### Population dynamics model

To assess the potential impact of adult-only, pupal-only or combined RIDL releases a model of wild *Aedes aegypti* population dynamics [13] was used. The rate of change in the number of females [F(t)] with respect to time is

(6) $$\frac{\mathrm{dF}}{\mathrm{dt}}=\mathrm{QF}\left(t-T\right)\exp\left[-\alpha {\left(\mathrm{EF}\left(t-T\right)\right)}^{\beta }\right]-\mathrm{\omega F}\left(t\right),$$

where Q is the birth rate (egg to adult) in the absence of any density-dependent larval effects, T is the mosquito development time, α the first larval density-dependent coefficient (set to determine the equilibrium number of females in the wild population in the absence of control), E is the female egg production rate, β the second larval density-dependent coefficient (set to = 1 throughout but included for generality and consistency with previous published studies using this model) and *ω* the adult mortality rate.

This model has been altered to include the effect of a RIDL release with late-acting lethality on the wild population dynamics [4], giving

(7) $$\frac{\mathrm{dF}}{\mathrm{dt}}=\left(\mathrm{QF}\left(t-T\right)\frac{F\left(t-T\right)}{F\left(t-T\right)+\mathrm{cD}\left(t-T\right)}\right)\exp\left[-\alpha {\left(\mathrm{EF}\left(t-T\right)\right)}^{\beta }\right]-\mathrm{\omega F}\left(t\right).$$

Parameters are as equation (6) with the addition of c, the mating competitiveness of RIDL males and D(t) the number of RIDL males at time *t*. In this instance the number of new females in the next generation is proportional to the ratio of wild to RIDL males in the population, adjusted for the relative competitiveness of RIDL males (*c*). A conservative value (*c* = 0.01) was assigned to male mating competitiveness and was assumed to be equal for all release types. The field estimate of mating competitiveness from the Cayman Islands was 0.059 (95% bootstrap CI 0.011-0.21) [6]. Other assumptions of this model include: a closed population, a 1:1 sex ratio of wild individuals in the absence of control, random mating, all individuals taking the same average time to progress through each life-stage [13,14] and releases being of 100% homozygous RIDL male insects carrying a late-acting lethal construct [4].

### Measuring performance

Measurement of the effectiveness of a given control approach involves a comparison between the population in the absence and presence of control. Previous studies have quantified the release effect for single releases [15], we employ a similar approach for multiple releases that allows a measurement of treatment effect relative to the wild population in the absence of control. The treated area under the curve (AUC) in the controlled population is

(8) $$\mathrm{Treated}\;\mathrm{AUC}=\frac{\int_{t_{1}}^{t_{n+400}}F_{c}\;\mathrm{dt}}{\int_{t_{1}}^{t_{n+400}}F_{0}\mathrm{dt}},$$

where *t*_*1*_ is the first release day, *t*_*n*_ the last release day, *F*_*c*_ the wild population of females in the presence of control and *F*_*0*_ the wild population in the absence of control. The control effect is measured 400 days after the last release to capture population recovery dynamics.

Where two different control methods or regimes are being compared, a relative measure of their respective effectiveness is used:

(9) $$\mathrm{Relative}\;\mathrm{effect}\;\mathrm{size}=\frac{\mathrm{Treated}\;\mathrm{AU}C_{a}}{\mathrm{Treated}\;\mathrm{AU}C_{b}},$$

where *a* denotes one control method or regime and *b*, the other. In this instance, values of relative effect size <1 occur when control *a* outperforms control *b*, values >1 occur when control *b* outperforms control *a*, values and relative effect size = 1 when the two approaches perform equally.

All simulations were programmed and implemented in the statistical program R [16] using the deSolve package [17], with default settings used for the differential equation solver. All parameters were set to the default values as specified in Table 1 throughout, unless otherwise stated.

**Table 1:** State variable and parameter definitions and default parameter values

**State variables**	**Definition**		
P	Number of Pupae		
A	Number of sexually immature adult males		
M	Number of sexually mature adult males		
R	Number of individuals that are recaptured		
F	Number of females in the wild population at time t		
D	Number of sexually mature RIDL adult males at time t		
**Wild parameter**	**Definition**	**value**	**ref**
Q	Number of offspring produced by each adult per day that survive to adulthood in the absence of density-dependent mortality	0.7	[14]
α	Density-dependent coefficient (set to determine the equilibrium number of females in the wild population in the absence of control)	1.21e^-05^	
β	Density-dependent coefficient	1	[13]
E	Female daily egg production	16	[13]
ω	Wild mosquito mortality rate (conservative value chosen) (days^-1^)	0.1	[18-20]
T	Mosquito generation time (days)	18.5	[13]
**RIDL parameter**	**Definition**	**value**	**ref**
*w*	Eclosion parameter (relates to the rate at which pupae eclose) (days^-1^)	1,2,4,8	estimated
*k*	Eclosion coefficient	1.01 × release size	estimated
δ	Mortality rate (days^-1^)	0.5	estimated
γ	Recapture rate (days^-1^)	0	estimated
c	RIDL male mating competitiveness (conservative value chosen)	0.01	[7,21,22]
σ	Sexual maturation rate (days^-1^)	1	[23]

### Comparing release methods and regimes

The efficacy of adult-only, pupal-only and combined release methods was tested on a large simulated wild population set to reach a stable equilibrium at 10,000 female *Ae. aegypti* in the absence of any control.

Simulations of releases were made for a number of scenarios varying the weekly production capacity (production from 2 × 10^7^ to 3.5 × 10^7^ males per week) and release frequencies (time between releases from 1 to 20 days). Individual release sizes were calculated as

(10) $$\begin{array}{l} \mathrm{release}\;\mathrm{size}=\frac{\mathrm{weekly}\;\mathrm{production}}{7}\\ \;\;\;\times \mathrm{days}\;\mathrm{between}\;\mathrm{release} \end{array}$$

Releases were started following 500 days of simulation without control and concluded after 100 days. All RIDL parameters were set as default (Table 1) except the pupal eclosion rate, *w*, which was varied for pupal-only release scenarios (*w* = 1, 2, 4, 8 days^-1^). For combined releases only one pupal eclosion rate (*w* = 2 days^-1^) was considered. This rate was at the lower end of estimates from the data and emphasised observed differences between adult and pupal dynamics. Adult and pupal releases were assumed to occur simultaneously and in a 1:1 ratio when in combination.

### Long-term suppression

The population dynamics model was further altered to assess the potential for various release strategies to successfully suppress the population in the long-term. A term that included stochastic increases in wild population numbers was added to represent external immigration pressures. Immigration was modelled as a random negative binomial process, with constant dispersion parameter, *z* = 1, and probability of success, *p* = 0.05. An independent immigration event was set to occur at the start of each day (mean = 1.9 individuals day^-1^, max = 148).

The potential benefits of adult-only, pupal-only or combined releases for the long-term maintenance of suppression of a population in the presence of immigration were studied. An initial intensive control effort was simulated to suppress the wild population down to a low level (100 days of adult-only releases every two days). The ability of adult-only, pupal-only or combined releases of low frequency (releases every 7 days) to maintain population suppression for 5 years was then examined. The effect of varying adult-to-pupae ratios in a combined release (ranging from all adults to all pupae) on the reduction in AUC was calculated. The ratio of adults to pupae used in the combined releases was set at the optimum, maximising the estimated level of suppression as a function of the adult to pupal ratio.

## Results

### Experimental data

From the four independent MRR experiments 46, 146, 32 and 56 marked adult males were recaptured in the field. Recaptures were conducted from one to thirteen days post-release. Recapture numbers peaked between two and four days post release, the latest recapture occurred on day nine. Pupal eclosion in the cage study was observed over a period of three days.

### Pupal dynamics model

**Figure 1:**
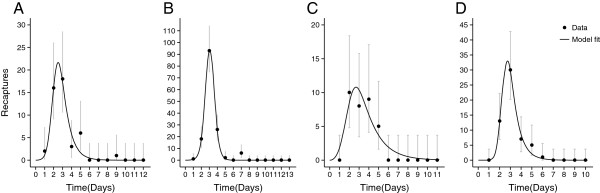
**Pupal MRR data and model fit.** The number of marked recaptured individuals with respect to time (with 95% Poisson confidence intervals for the underlying rate) and estimated model fit for four MRR experiments **(A-D)**.

**Figure 2:**
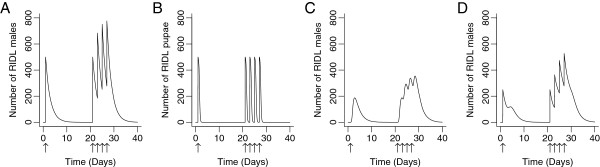
**Multiple, pulsed releases on RIDL insects.** Examples of pulsed releases of **A)** adults only and **B)** pupae only. The resulting, **C)** sexually mature adult males from a pupal release and **D)** sexually mature adults from a combined release. Arrows denote release days, all parameter values as default, *w* = 2 days^-1^. Frequent releases show a cumulative effect.

Model fit of the pupal dynamics model to pupal MRR data from Grand Cayman was qualitatively good with predicted values falling within the 95% confidence intervals for all but two time points (Figure 1) allowing estimates of mortality and recapture rates to be made (Table 2). The pupal eclosion model given in Equation 1 reproduced the observed trends in pupal eclosion data but had an associated lag (Additional file 2: Appendix 2). When explicitly modelled, this lag, attributed to time spent within the release device, was estimated as being between 12 and 18 hours. The pupal dynamics model and the adult model were used to simulate pulsed releases of RIDL males (Figure 2).

**Table 2:** Estimated parameter values for pupal MRR data

**Release date**	**Number released**	**Mortality rate (*δ *** **in days**^**-1**^**)**	**Proportion that survive from one day to the next**	**Recapture rate (*γ *** **in days**^**-1**^**)**	**Eclosion rate (w in days**^**-1**^**)**
22/09/2010	19624	1.25	0.28	0.0027	3.82
24/09/2010	38968	6.95	0.001	0.025	3.40
08/10/2010	16673	0.64	0.53	0.0014	2.22
13/10/2010	22702	1.40	0.25	0.0034	5.86

### Release method comparisons

**Figure 3:**
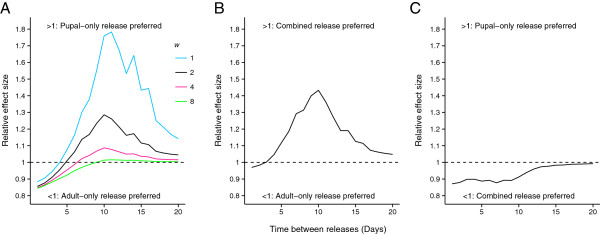
**The relative effectiveness of releasing different life stages.** The relative effect size (Eq. 9) of **A)** adult-only against pupal-only (*w* = 1,2,4,8 days^-1^), **B)** adult-only against combined releases (*w* = 2 days^-1^) and **C)** combined against pupal-only release (*w* = 2 days^-1^) for a range of release frequencies . All parameter values (except *w*) as default, production = 3.5 × 10^7^ males per week.

Trends in the relative effect of different release methods remained consistent for the range of release sizes and timings considered, therefore, results assuming a production = 3.5 × 10^7^ males week^-1^ are shown (Figure 3). The maximum departure from 1 of the relative effect size measure observed was 0.78. For all comparisons the relative effect size tended towards 1 with less frequent releases (time between releases of >15 days).

Adult-only releases were universally more effective compared with the four pupal-only release scenarios and combined releases when release frequency was every 4 days or more frequent. The relative performance of adult-only releases was greatest when releases occurred daily. The relative performance of pupal-only releases was strongly influenced by the rate of eclosion (*w*). For slow eclosion rates (*w* = 1, 2 days^-1^) pupal-only releases strongly outperformed adult-only releases when releases were at least 5 days apart. The strength of this relative advantage waned with increasing eclosion rates. The highest rate of pupal eclosion considered (*w* = 8 days^-1^) saw nearly all advantages of pupal-only releases over adult-only releases at infrequent release periods diminished. Combined release methods (*w* = 2 days^-1^) were more effective than adult-only release methods for releases at least 4 days apart and outperformed all pupal-only releases (*w* = 2 days^-1^) considered.

**Figure 4:**
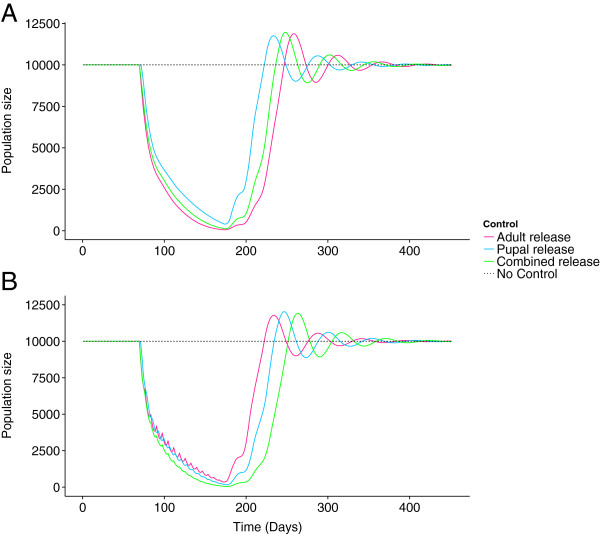
**Examples of control with frequent and infrequent releases.***Ae. aegypti* population dynamics in the absence of control (dotted line) and under adult-only, pupal-only and combined RIDL release regimes (pink, blue and green lines respectively). With **A)** frequent releases (time between releases = 1 day) adult-only releases achieve the best levels of population suppression. With **B)** infrequent releases (time between releases = 7 days) wild population recovery between releases can be observed and is most apparent with adult-only releases which are outperformed by combined and pupal-only releases. All parameter values as default, *w* = 2 days^-1^.

Examples of how these relative effect sizes may translate to wild-population suppression in a RIDL control programme are shown in Figure 4. Here, two scenarios are considered, one where releases are daily (7 × 1,000,000 release week^-1^) and one where releases are every 7 days (1 × 7,000,000 release week^-1^). The release programme is simulated for a period of 100 days. Daily adult-only releases outperform daily pupal-only releases and, marginally outperform the combined releases. Infrequent (every 7 days) pupal-only releases are marginally more successful at suppressing the wild population than adult-only releases at the same frequency, whilst the combined releases perform most effectively.

### Long-term suppression

**Figure 5:**
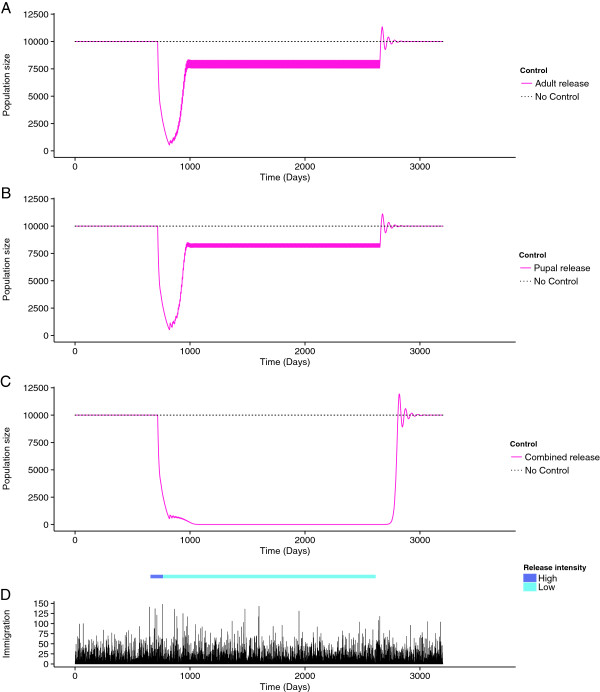
**The long term maintenance of suppression of a simulated wild population.** Long-term suppression with infrequent **A)** adult-only, **B)** pupal-only (*w* = 2 days^-1^), and **C)** combined releases, (*w* = 2 days^-1^), following population suppression by an intensive release of adults. Combined releases successfully maintain suppression in the long-term with low intensity releases adult- and pupal-only releases do not. Releases are every day and every seven days at high and low release intensities respectively. The model includes **D)** immigration events into the simulated population.

For a release frequency of every seven days the optimum ratio of pupae to adults was close to 1:1 (55% pupae, 45% adults) (Additional file 3: Appendix 3). Scenarios with infrequent releases indicated that combined releases may be a more efficient way of maintaining suppression than adult- or pupal-only releases in the long-term. Maintenance of suppression was achieved when releasing 1.9 million individuals (1,387,000 pupae + 513,000 adults), every seven days in combined releases. Adult- or pupal-only releases at these numbers failed to maintain suppression in the target population (Figure 5) requiring releases of 2.8 and 2.7 million individuals per week respectively to maintain suppression.

## Discussion

This study makes use of large-scale pupal MRR data to inform models that allow comparisons between adult-only, pupal-only and combined releases of RIDL *Ae. aegypti* over a range of scenarios. Adult-only releases are most beneficial when releases are frequent, whilst pupal-only and combined releases may outperform adult-only releases in scenarios when releases are less frequent. Potential approaches to maintaining long-term suppression of the vector population have been explored. Combined releases can provide increased effectiveness for a long-term vector control programme.

The pupal release model provides a method by which adult male numbers from RIDL pupal-only pulsed releases can be simulated over time. The fit of the model-predicted recaptures to recapture data was good (Figure 1); however, there were differences between observed and predicted pupal numbers over time (Additional file 2: Appendix 2, A-D). The predicted pupal numbers show similar but lagged trends to numbers predicted from eclosion experiments. One potential cause for this lag would be eclosed males resting in the pupal release device before exiting. The number of males predicted by the model would be those functional males that had exited, not eclosed males within the release device. A model explicitly including this lag improved the fit to pupal eclosion data (Additional file 2: Appendix 2, E-H), at the expense of reduced fit to the recapture data. The more simple model and the sigmoid functional form of pupal eclosion was chosen for all simulations as they provide a superior fit to recapture data. Assuming recapture rates were constant, both models predicted similar numbers of mature males over time, the critical factor for control efficacy. A second potential explanation may be disparities in the recapture rates of sexually immature and sexually mature males. A similar lag may be explained by newly eclosed males being less likely to be recaptured. Throughout this study the use of appropriate field data as the basis of model design and utilisation has been championed. Even in this scenario, where very large scale MRR experiments were used to parameterise the model, there is scope for further field studies to improve the understanding of the early-stage dynamics of a pupal release. This emphasises the iterative process by which data inform models that in turn can be used to influence the design and direction of future field studies.

The ability of adult-only releases to consistently outperform both pupal-only and combined releases at high release frequencies is a very clear outcome of this analysis. The benefits of frequently releasing adults have also been shown in other studies [15]; to date, most programmes using RIDL *Ae. aegypti* have involved relatively frequent adult-only releases of males, e.g. three releases per week [6]. Adult-only releases introduce males that are already sexually mature. Pupal-only releases will always be disadvantaged in comparison as newly eclosed males will almost certainly experience higher mortality rates in the period it takes them to sexually mature in the field than individuals that have sexually matured prior to release. Frequent releases of adults perform well due to the initial spike in RIDL numbers immediately after release. Numbers remain relatively high until the next release (e.g. the next day), where a new pulse of sexually active RIDL males is introduced into the population allowing very high densities of sexually active RIDL males to be maintained over time. A high RIDL-to-wild-male ratio increases the chances of a wild female mating a RIDL male resulting in increased numbers of infertile matings, thus improving control. When releases are less frequent the effectiveness of adult-only releases is reduced. Troughs in RIDL numbers between releases allow wild population recovery, reducing the suppressive effect (Figure 4B).

The peak in sexually active males from a pupal-only release is delayed and prolonged compared with that from an adult-only release (Figure 2). This is best taken advantage of with less frequent releases of RIDL insects. Here, the less peaked distribution of sexually mature adults over time provides better coverage of RIDL males in the population when releases occur less often. Our results reflect this, with pupal-only releases outperforming adult-only releases when the time between releases becomes greater (Figure 3A).

The relative performance of a pupal release is highly dependent on the rate at which pupae eclose. The relative benefit of pupal releases decreases with increasing pupal eclosion rates when releases are infrequent. Increasing eclosion rates produce a shorter and more intense pulse of adult insects into the population, akin to an adult-only release. The actual pupal eclosion rates may be highly dependent on external variables, such as temperature [24-26]. The lowest estimate of *w*, 2.22 (Table 2), would lead to considerably different dynamics compared with an adult release, however, eclosion rates as low as 1 day^-1^ are unlikely without some external manipulation. The ability to predict and manipulate the eclosion rates for a given target area may significantly affect the performance of pupal-only releases compared with the adult-only release method. These effects may be even more pronounced if earlier life-stages, such as eggs [27], were distributed.

Combined releases have the potential to benefit from both the initial peak produced by the adult component of the release as well as the secondary peak of RIDL males from the pupal component. Whilst undoubtedly being logistically more challenging, combined releases outperformed both pupal-only and adult-only releases for the majority of scenarios considered. Combined releases show the strongest suppression of wild population recovery between releases when time between releases was seven days (Figure 4B). Combined releases are only marginally outperformed by adult-only releases when release frequency is < every 4 days (Figure 3B).

It is important to note that throughout these analyses any spatial element has been omitted. This approach was chosen to allow clear comparisons between the release types examined. Field and suppression programme release of RIDL individuals, be it pupal or adult, are likely to encounter spatial heterogeneity and metapopulations, which can lead to reduced efficacy of a RIDL-based approach [27]. All of the approaches considered have benefits and drawbacks unrelated to population dynamics that will also determine their feasibility and use. Production costs for adult-only releases would include the storage and maintenance of eclosing adults, an additional stage not required for pupal-only releases. The distribution of adult-only releases may be logistically the most simple, with releases potentially able to be performed from a moving vehicle or even aircraft. Pupal release would involve the distribution of pupal release devices and may be prone to problems of disturbance, theft or predation. However, pupae are a more robust life stage than adults making them more amenable to long-distance travel between production facilities and release sites. The gradual appearance of adult mosquitoes in the control area from pupal releases may also reduce the perception of public nuisance.

Releases of RIDL *Ae. aegypti* have been shown to successfully suppress wild populations in a short period of time [6]. To maximise the potential vector control and public health benefits of a vector control programme such suppression must be maintained in the long-term. Maintenance of suppression must be conducted in a cost-effective manner in the face of immigration pressures from external populations. When population numbers have been driven to very low levels, random events may have relatively large impacts on population dynamics. We included this stochastic term into the model to emphasise the potential for single, sporadic immigration events to disrupt maintenance of suppression of a wild population. Optimising low-intensity maintenance releases of RIDL insects will be vital to achieve the goal of long-term suppression. Low-frequency releases of a combination of adults and pupae may be the most effective method of maintaining suppression in the long-term (Figure 5C). This method allows fewer insects to be released at low-frequency compared with adult- or pupal-only releases. These benefits could outweigh the disadvantages of the logistical demands of a combined release and help to provide an optimal cost-effective, long-term solution.

## Conclusions

This study demonstrates the process by which field data can be successfully used to design models to inform future studies and practical approaches. Parameterised models show that adult-only, pupal-only and combined RIDL releases all demonstrate a good ability to supress a simulated wild population of *Ae. aegypti.* When releases are frequent, adult-only releases are superior to pupal-only and combined releases and have in their favour more simple logistical implementation in the field. Under certain circumstances, such as when releases are more infrequent, pupal-only and combined releases can outperform adult-only releases. The relative benefit of using combined releases when releasing infrequently suggests they could be utilised to maintain long-term suppression in a sustainable manner.

## Abbreviations

RIDL: The release of insects carrying a dominant lethal; MRR: Mark-release-recapture; DHF: Dengue haemorrhagic fever; DSS: Dengue shock syndrome; SIT: Sterile insect technique; AUC: Area under the curve.

## Competing interests

Authors affiliated with Oxitec Ltd are staff or students of Oxitec and have employment, studentship support and/or equity interest in Oxitec. Oxitec and the University of Oxford own intellectual property related to the subject matter of this study. All other authors declare no competing interests.

## Authors’ contributions

PW performed the modelling and statistical analysis and wrote the manuscript. AH, LA, ARM, SAM, JS and NR all participated in the original field trial. CAD advised on statistics and modelling. CAD, LA and ARM all helped to draft the manuscript. All authors read and approved the final manuscript.

## Supplementary Material

Additional file 1 Appendix 1. Click here for file

Additional file 2 **Appendix 2.** Model validation using pupal eclosion data. Click here for file

Additional file 3 **Appendix 3.** Optimising the adult-to-pupae ratio. Click here for file
