# Supplementary material for: Genetic control of Aedes aegypti: data-driven modelling to assess the effect of releasing different life stages and the potential for long-term suppression
Source: Parasit Vectors. 2014 Feb 13;7:68. doi: 10.1186/1756-3305-7-68 (PMC3944930; doi:10.1186/1756-3305-7-68)
Supplement: Additional file 1 — Appendix 1. [file 1756-3305-7-68-S1.doc]

### Additional file 1: Appendix 1

A more complex model of pupal dynamics was formulated which included an additional compartment to represent eclosed males that had not left the pupal release device. In this instance, equation 1 remains

|  | 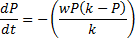 | A1. |
| --- | --- | --- |

The additional compartment represents the rate of change in the number of males that have eclosed, but not exited the release device (L)

|  | 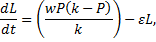 | A2. |
| --- | --- | --- |

where *ε*, represents the rate that eclosed males exit the release device (*ε^-1^* being the mean duration of rest within the release device). The rate of change in the number of sexually immature adults that have exited the release device now becomes

|  | 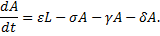 | A3. |
| --- | --- | --- |

The rate of change in the number of individuals that are sexually mature adults (M) with respect to time is

|  | 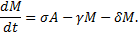 | A4. |
| --- | --- | --- |

The rate of change in the number of recaptured individuals is therefore dependent on the rates of recapture of both sexually immature and mature adults

|  | 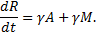 | A5. |
| --- | --- | --- |
