# Supplementary material for: Genetic control of Aedes aegypti: data-driven modelling to assess the effect of releasing different life stages and the potential for long-term suppression
Source: Parasit Vectors. 2014 Feb 13;7:68. doi: 10.1186/1756-3305-7-68 (PMC3944930; doi:10.1186/1756-3305-7-68)
Supplement: Additional file 2 — Appendix 2. Model validation using pupal eclosion data. [file 1756-3305-7-68-S2.doc]

**Additional file 2: Appendix 2. Model validation using pupal eclosion data**. Pupal eclosion data (with 95% confidence intervals for the underlying rate) and predicted numbers from models fitted to recapture data for A-D) simple model (eq. 1-4), E-H) the model including a lagged exit from the release device (eq. A1-A4).
