# Supplementary material for: Genetic control of Aedes aegypti: data-driven modelling to assess the effect of releasing different life stages and the potential for long-term suppression
Source: Parasit Vectors. 2014 Feb 13;7:68. doi: 10.1186/1756-3305-7-68 (PMC3944930; doi:10.1186/1756-3305-7-68)
Supplement: Additional file 3 — Appendix 3. Optimising the adult-to-pupae ratio. [file 1756-3305-7-68-S3.doc]

**Additional file 3: Appendix 3. Optimising the adult-to-pupae ratio.** The level of suppression of a wild population achieved as a function of varying adult-to-pupae ratios in a combined release. The optimum ratio of pupae to adults for release every seven days is (55% pupae, 45% adults).
